# An Integrated Pharmacophore/Docking/3D-QSAR Approach to Screening a Large Library of Products in Search of Future Botulinum Neurotoxin A Inhibitors

**DOI:** 10.3390/ijms21249470

**Published:** 2020-12-12

**Authors:** Davide Gentile, Giuseppe Floresta, Vincenzo Patamia, Rita Chiaramonte, Giulia Letizia Mauro, Antonio Rescifina, Michele Vecchio

**Affiliations:** 1 Department of Drug Sciences, University of Catania, V.le A. Doria, 95125 Catania, Italy; davide.gentile@unict.it (D.G.); giuseppe.floresta@unict.it (G.F.); vincenzo.patamia@unict.it (V.P.); 2 Department of Biomedical and Biotechnological Sciences, Section of Pharmacology, University of Catania, Via S. Sofia 67, 95123 Catania, Italy; ritachiaramd@gmail.com; 3 Department of Surgery, Oncology, and Stomatology, University of Palermo, Via Liborio Giuffrè 5, 90127 Palermo, Italy; giulia.letiziamauro@unipa.it

**Keywords:** botulinum neurotoxin A, virtual screening, docking, 3D-QSAR, molecular dynamics

## Abstract

Botulinum toxins are neurotoxins produced by *Clostridium botulinum*. This toxin can be lethal for humans as a cause of botulism; however, in small doses, the same toxin is used to treat different conditions. Even if the therapeutic doses are effective and safe, the adverse reactions could be local and could unmask a subclinical impairment of neuromuscular transmissions. There are not many cases of adverse events in the literature; however, it is possible that sometimes they do not occur as they are transient and, if they do occur, there is no possibility of a cure other than to wait for the pharmacological effect to end. Inhibition of botulinum neurotoxin type A (BoNT/A) effects is a strategy for treating botulism as it can provide an effective post-exposure remedy. In this paper, 13,592,287 compounds were screened through a pharmacophore filter, a 3D-QSAR model, and a virtual screening; then, the compounds with the best affinity were selected. Molecular dynamics simulation studies on the first four compounds predicted to be the most active were conducted to verify that the poses foreseen by the docking were stable. This approach allowed us to identify compounds with a calculated inhibitory activity in the range of 316–500 nM.

## 1. Introduction

Botulinum toxins are potent neurotoxins produced by Clostridium botulinum, causing a potentially life-threatening neuroparalytic disease that is generally acquired by ingesting contaminated food. The action mechanism consists of the inhibition of acetylcholine release by the presynaptic nerve in the neuromuscular junctions, with consequent effects ranging from generalized muscle paralysis to respiratory arrest. Seven antigenically distinct botulinum neurotoxins are known (serotypes A, B, C, D, E, F, and G), but serotypes A, B, E, and F have been reported to be the main culprits of botulism in humans. The lethal doses of botulinum neurotoxin type A (BoNT/A) for a human being of 70 kg are estimated to be 0.9 µg by inhalation and 70 µg by ingestion [1]. Despite its toxicity, serotype A is often used for a variety of neurological disorders, such as dystonia, blepharospasm, hyperhidrosis, neurogenic bladder, strabismus, chronic migraine, and facial hemispasm; in cosmetic applications (marketed as Botox) in order to temporarily reduce facial frown lines; and, at high doses, even for spasticity. Although it seems that the use of high doses [2] or repeated-dose [3] of botulinum toxin is safe and effective, the possibility of developing a form of iatrogenic, systemic, or focal botulism [4] cannot be totally excluded during clinical practice. In fact, the adverse reactions could be local, such as ptosis in blepharospasm treatment, or systemic, such as general weakness, flulike symptoms, allergic reactions, bulbar symptoms, dysphagia, and respiratory distress [4,5,6]. Moreover, injections of the toxin could unmask a subclinical impairment of neuromuscular transmissions, such as myasthenia gravis [7]. The duration of the clinical effects of the toxin and the eventual adverse events is usually 2–3 months after injection.

Despite the fact that there are only a few cases of iatrogenic botulism reported in the literature in terms of systemic diffusion of the drug characterized by generalized weakness or in terms of district diffusion in muscles close to the inoculated ones characterized by focal weakness, it is likely that many cases of weakness are not described in the literature as they are transient. A transient adverse effect, in all cases, disables the subject. Having a drug available that limits the duration of the adverse event, for which, in current day, the only remedy is to wait for the effect to pass by itself, would be useful to many doctors who practice this therapy and, above all, useful to the patient, who is often disabled. This period of waiting, due to adverse effects, can aggravate a patient’s disability.

It is always useful to try to counteract the adverse events of drugs, even if they are rare. This paper emphasizes the unfortunate reality that the only weapons to counteract the adverse effect of a drug administered improperly are waiting and, if necessary, supporting vital functions.

Structurally, BoNT/A consists of a light chain (LC) and a heavy chain linked by a disulfide bond. The LC is a zinc endopeptidase that specifically cleaves SNAP-25 (synaptosomal-associated protein 25), a neuronal protein required for acetylcholine release [8]. The LC of BoNT/A (LC/A) has been a target for developing small-molecule inhibitors of the toxin [9]. However, developing potent inhibitors is tricky, because after the substrate of LC/A binds the protein, they are characterized by an unusually large substrate enzyme interface area of 4840 Å^2^ that would require a complex structure inhibitor. Such inhibitors are expensive to synthesize and challenging to design. In this regard, it could be tempting to seek molecules that have functional groups capable of blocking the toxin as potential inhibitors.

Moreover, a possible natural inhibitor would avoid lengthy and expensive synthesis processes. Despite the numerous disclosures of BoNT/A inhibitors at the LC active site, the design of submicromolar inhibitors remains difficult. This is also due to the highly flexible nature of the enzyme. In recent years, several types of BoNT/A inhibitors that directly affect the LC endosomal translocation or LC endopeptidase have been developed. Zinc-chelating molecules have been shown to be privileged inhibitor scaffolds against zinc metalloproteases; for this reason, hydroxamates have been widely studied as LC/A inhibitors [10]. Exploiting computer-aided molecular design (CAMD), this article attempts to overcome the unfortunate reality that the only weapons to counteract the negative effect of a drug administered improperly are waiting and searching for an effective antidote, even if the adverse events reported appear to be rare. Nowadays, CAMD and computational chemistry are essential aspects of drug design and have been used to follow atom-by-atom interaction and reactivity [11,12]. From hit identification to lead optimization and beyond, approaches such as structure-based, ligand-based, and virtual screening are widely used by our research group to support drug discovery efforts [13,14]. Generally, these methods can be divided into structure-based and ligand-based drug design. In the first category, using available 3D structural and other relevant biological information concerning the target protein, the binding energy of small molecule inhibitors is calculated, and the chemical structures are modified accordingly with the target information. Often, the 3D structural information of the target protein is not available, and/or there is no good template for homology modeling; in this case, it is more convenient to use the ligand-based method. A series of descriptors are calculated from the dataset obtained, and QSAR equations are derived from building a pharmacophore model that is used to suggest new compounds with improved activity. In some other cases, the two different structures and ligand-based methodologies can be joined together, as we previously did and as we present in this paper, to filter a significant number of compounds [15,16].

## 2. Results and Discussion

### 2.1. Pharmacophore Model

Previously, several 3D search queries were developed for the identification of LC/A inhibitors [17,18,19,20,21]. A model called “3-zone pharmacophore” was used for the discovery of four non-Zn(II) coordinating inhibitors with moderate in vitro activity [19]; this approach identifies zone 1 as an aromatic component, zone 2 as a cationic group, and zone 3 as a hydrophobic moiety. A flexible alkyl linker connects all pharmacophore zones. A steric and electronic improvement of zone 3 led to the discovery of a new inhibitor with a *K*_i_ value of about 600 nM [20]. The same authors implemented the 3-zone pharmacophore model by adding a fourth aromatic zone (for example, indoles, phenyls, pyridines, etc.) [21]. This new query allowed the identification of an inhibitor possessing a *K*_i_ value of 572 nM. Unfortunately, none of these models succeeded in finding compounds more potent than the co-crystallized (2*E*)-3-(2,4-dichlorophenyl)-*N*-hydroxyacrylamide (DCNHA) [22] (*K*_i_ = 300.0, [23]) or 2-(adamantan-1-yl)-*N*-hydroxyacetamide [24] (*K*_i_ = 460.0, [24]). Thus, considering the moderate results obtained from the 3-zone pharmacophore models and the remarkable ability of hydroxamates to chelate the zinc atom within the active site of LC/A, we decided to develop a pharmacophore model in agreement with the main characteristics of the most potent crystallized inhibitor DCNHA, while being aware that this limits the possibility of finding structurally new molecules.

To identify new potent inhibitors of LC/A, the first step of our rational approach was building a 3D pharmacophore model of the enzymatic cavity starting from the crystallized structure of the LC/A complex with DCNHA (ID PDB: 2IMA). The binding mode of the hydroxamic acids was rationalized by the X-ray data, demonstrating that this series of inhibitors was able to occupy the catalytic cavity of the enzyme and coordinate the zinc atom. In particular, the DCNHA binds with the cinnamyl region oriented towards the Asp370 residue, which forms a wall at one end of the binding cavity and forms part of the *β*-exosite [25]. The hydroxyl oxygen of the hydroxylated moiety coordinates the catalytic ion Zn^2+^, while Tyr366 forms a hydrogen bond with the carbonyl oxygen. Glu224, typical of many zinc proteases, forms an H bond with the hydroxyl oxygen of the hydroxamate, while the amide nitrogen hydroxide forms a hydrogen bond with the carbonyl oxygen of the backbone of Phe163.

**Figure 1:**
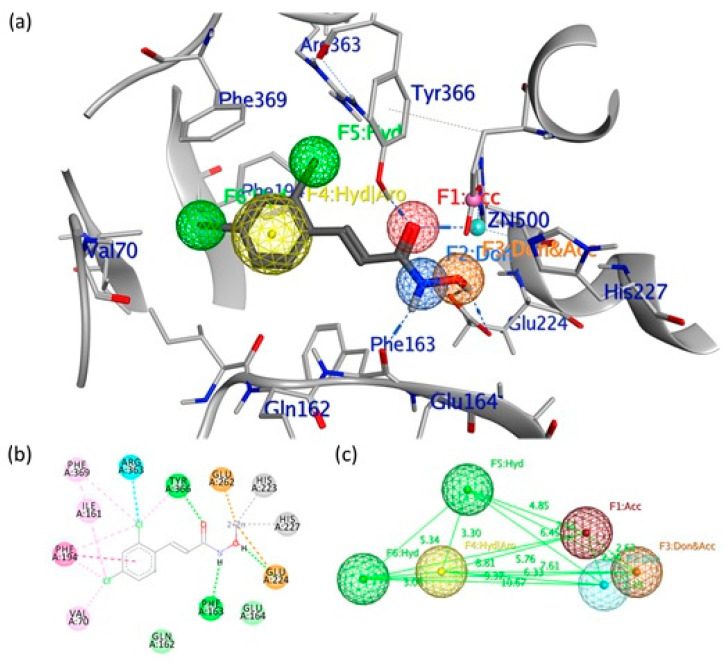
(**a**) Pharmacophore model generated by the Pharmit server, including hydrogen bond donors (Don) (blue/orange spheres), negatively charged oxygen atom to represent a hydrogen bond acceptor (Acc) (red sphere), the hydrophobic center (Hyd) (green sphere) and the aromatic center (yellow sphere). (**b**) Binding site interactions between the light chain of botulinum neurotoxin type A (LC/A) binding pocket and the co-crystallized (2*E*)-3-(2,4-dichlorophenyl)-*N*-hydroxyacrylamide (DCNHA). (**c**) 3D spatial distribution of the six pharmacophore features.

Based on the co-crystal structure obtained from LC/A in complex with the DCNHA inhibitor, we generated the structure-based pharmacophore model (Figure 1) via the Pharmit server [26], which provides both pharmacophore and molecular shape search modalities as well as a ranking of results by energy minimization.

In particular, the pharmacophoric model obtained for the crystallized ligand includes the following characteristics: the hydroxamic portion, represented by two H bond donors (Don) and an H bond acceptor (Acc), while in the aromatic portion, there is a hydrophobic/aromatic center (Hyd/Aro) and two hydrophobic interactions (Hyd). The essential characteristics of the generated pharmacophore model included two hydrogen bond donors (pharmacophore areas F2 and F3, Figure 1) located in an opportune position to interact with residues Phe163 and Glu224, respectively; one hydrogen bond acceptor feature (pharmacophore area F1, Figure 1) that is positioned to interact with Tyr366 residue; one aromatic/hydrophobic center feature (pharmacophore area F4, Figure 1); and two hydrophobic points (pharmacophore areas F5 and F6, Figure 1) that are associated with the interactions of the phenyl group of the inhibitor situated in the hydrophobic pocket. The aromatic/hydrophobic region of the inhibitor is located at a distance of 6.33 Å from the hydrophilic center (hydroxamic group). At the same time, the stereo-electronic characteristics of the generated pharmacophoric model are fundamental for the search for selective inhibitors for the active site of LC/A.

The generated pharmacophore model was used to filter a vast library of natural and synthetic compounds belonging to four databases: Marine Natural Product (MNP), Super Natural Product (SNP), ZINC and Molport Natural (MPN) for a total of 13,592,287 compounds and 126,606,488 conformers. The chemical database was screened against the pharmacophore model to find the best matches in terms of root mean square distance (RMSD) between pharmacophore query features and corresponding ligand points. The compounds with the lowest RMSD value (less than 2) were selected for further minimization. Single conformers with lower RMSD and max score (Δ*G*_B_) below −7 kcal/mol have been used for more accurate docking studies. At the same time, to validate the entire procedure, we seeded the dataset mentioned above with 18 compounds (the CONTROL dataset) chosen among the most potent LC/A inhibitors whose *K*_i_ is known; this dataset consists of compounds with a *K*_i_ ≤ 3 mM and an MW ≤ 700 D (Table S1).

**Figure 2:**
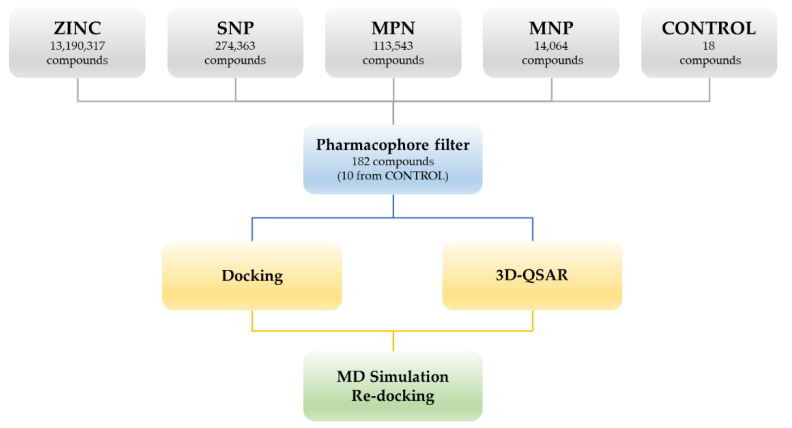
Workflow of the adopted molecular modeling procedure.

The 182 structures filtered and selected according to the pharmacophore descriptors (10 come from the CONTROL dataset) were used to perform docking studies and 3D-QSAR analysis (Tables S1 and S2). The workflow of the adopted molecular modeling procedure is reported in Figure 2.

### 2.2. 3 D-QSAR Analysis

**Figure 3:**
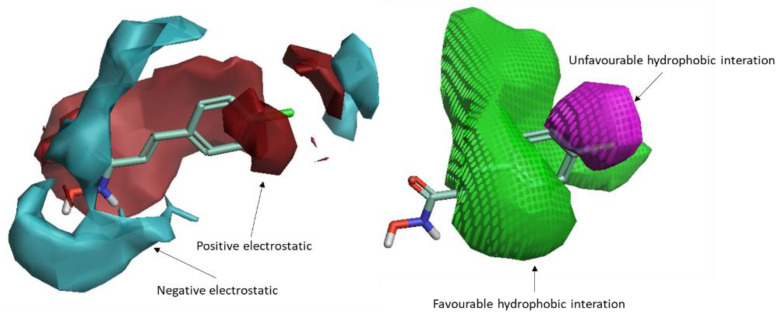
The 3D-QSAR model map is superimposed to 4-chlorocinnamic hydroxamate (originally used as a reference compound for the alignment). The positive field regions are red, and the negative ones are blue. Favorable shape/hydrophobic regions are green, and unfavorable shape/hydrophobic regions are violet.

A 3D-QSAR ligand-based filter (Figure 3) was then used to score the filtered dataset of 182 compounds. To this aim, all of the molecules were aligned to our previously published 3D-QSAR model for the LC/A protein [27], as described in the experimental section. After the alignment, the molecules were scored. In this procedure, the field points [28] of each molecule are considered, and it is assumed that molecules with similar field points would have similar interaction with biological targets, i.e., similar biological activity.

The calculated and compared field points were electrostatic, van der Waals, and hydrophobic potentials. The list of scored compounds, along with their calculated pIC_50_ and distance to the model parameter, is reported in Table S2. The best-scored compounds are reported in Table 1, excluding those belonging to the control dataset. All of them resulted in an optimal distance to the original model; this means that the original model well describes the molecules, i.e., most of the relevant features of the molecules were present in the training set of the 3D-QSAR model, and hence that the predicted activity based on the alignment with the original model is reliable. The validation of the model was previously performed with 123 (training set; leave-many-out q^2^ = 0.72, leave-one-out q^2^ = 0.73) plus 31 (test set; r^2^ = 0.68) molecules by calculating their activity and comparing the data with the experimentally measured ones [27]. An ulterior validation was made here and is reported in Section 2.4.

### 2.3. Docking and Molecular Dynamics Simulations

The compounds selected from the pharmacophore model were also subjected to docking experiments using Autodock4 implemented in YASARA software [29].

**Figure 4:**
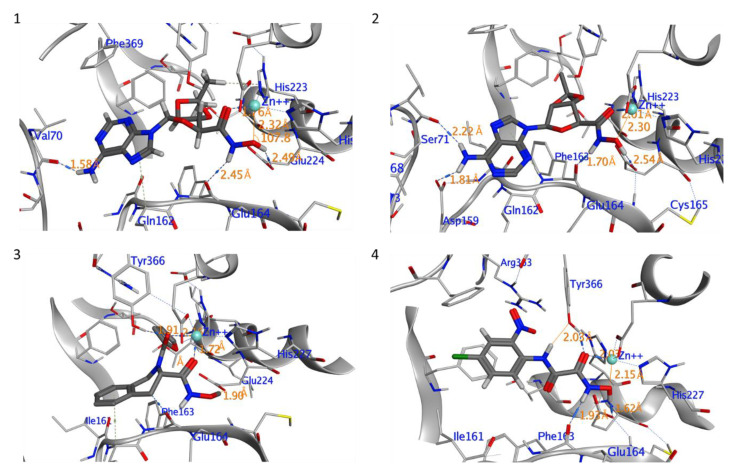
Interaction profile of the best-docked poses for compounds **1**–**4**.

The ten compounds with the best-predicted mean activity resulting from both 3D-QSAR and docking methodologies are reported in Table 1, excluding those belonging to the control dataset, together with the mean of the predicted pIC_50_ and p*K*_i_ values. Interestingly, among the different series, there are three diastereoisomers (compounds **1**, **2**, and **7**), while compound **4** is structurally similar to crystallized hydroxamate inhibitors. All compounds have a mean predicted value (used as a consensus score of the combined screening approach) between 6.2 and 6.5, and the 3D representations of the best-docked poses of the first four compounds are depicted in Figure 4.

To study the ligand stability in the active pocket, MD simulations were performed on the first four structures, and a re-docking study was performed on the last 3 ns-averaged structures (Table 1). RMSD analysis was performed on protein backbone atoms (both in the complex and free state), and the average RMSD values obtained for the ligand-bound proteins (i.e., the protein–inhibitor complex) and ligand are reported in Figures S1–S4.

As observed in other structures of LC/A complexes with hydroxamate inhibitors, ZINC5008970, the hydroxamate moiety, binds the Zn^2+^ ion in a bidentate manner with the carbonyl and hydroxyl oxygen atoms (1.76 and 2.32 Å, respectively) and a D–H–A angle of 107.8°. Hydroxylated nitrogen engages in a hydrogen bond interaction with the carbonyl of the main chain of Phe163, part of a *β*-filament that forms a wall of the active site (Figure 4, **1**). The purine group occupies a large hydrophobic pocket consisting of the lateral chains of Ile161, Phe163, Phe194, and Phe369, establishing CH–*π* interactions with Gln162 and an H bond with the carbonyl oxygen of Val70. The ketal group is inserted inside a sub-pocket, establishing CH–*π* interactions with His223. The MD simulation confirms that the stability of the ligand is reached after 4 ns and the protein structure after 1 ns. The potential energy of the system is stable throughout the simulation.

Unlike the other structures, ZINC53720402 is the only compound to form bidentate coordination with the zinc atom present in the active site through two atoms of the carbonyl oxygen of amide and carboxamide groups (Figure 4, **3**). The ligand is further stabilized by the H bond with Tyr366 and the *π*–*π* and alkyl–pi interactions with His223 and Gln162. The overall RMSD for the protein system appeared to have reached equilibrium after 800 ps and the stabilization of the protein–ligand complex after 1 ns, keeping interactions constant within the active site.

ZINC 5008966, the diastereoisomer of ZINC5008970, has a docking pose very similar to the first one (Figure 4, **2**). The difference in the free energies of binding between the two compounds is 0.3 kcal/mol in favor of ZINC5008970, despite an additional H bond with Asp159. Probably, the less unfavorable contacts of the ketal group inside the sub-pocket improve the affinity of the ligand. In fact, during the MD simulation, the eight-term bridge cycle tends to move away from the active site, breaking the *π*–*π* interactions between the aromatic area of the ligand and Phe369. RMSD of ZINC5008970 in MD simulation has a lower value than ZINC 5008966 of 1.2 Å, confirming the best stability of the docking pose.

**Figure 5:**
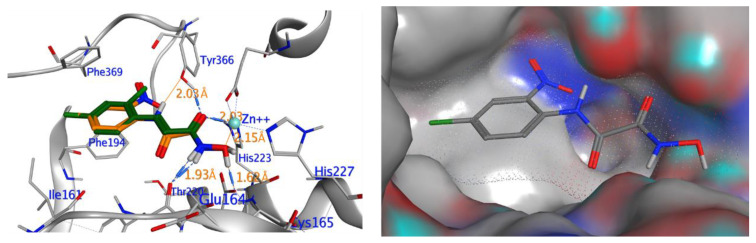
Interaction profile of the best-docked poses for compounds **4** (orange, stick model) and crystallized ligand (green, stick model) (**left**). View of **4** inside a binding pocket in electrostatic potential surface representation (**right**).

Among the compounds with the best activity, ZINC5729284, a commercially available chemical, is very similar to the known cinnamyl hydroxamate inhibitors. In fact, the hydroxamate moiety of ZINC5729284 identically coordinates Zn^2+^, while the amide hydrogen further stabilizes the pose of the ligand employing the H bond with Tyr366 at 2.03 Å (Figure 4, **4**). The aromatic moiety of the ligand settles in the hydrophobic region of the binding pocket, establishing *π*–*π* interaction with Phe194. The low RMSD value and the good stability of the ligand pose during the MD simulation confirm the excellent stability of the ligand–protein complex. Interestingly, the nitro group (negative electron density) fits inside a sub-pocket with a positive electron density due to Arg363, establishing electrostatic interactions that improve the affinity of this inhibitor (Figure 5).

The hydroxyl group of the hydroxamic functionality of compounds **1**, **2**, and **4** forms an H bond with the Glu224 residue, which is stable throughout the MD simulation (Figure S5); the H bond of compound **4** shows greater stability with an average distance of 1.96 Å, while compound **1** undergoes more significant fluctuations despite the lower average distance (1.88 Å). The energies of binding (calculated by the md_analyzebindenergy macro implemented in the YASARA software), including the time average, along MD simulation trajectories were employed to assess the strength of the interactions between the ligand and the binding pocket in the dynamic environment. Compound **4** shows a more stable fluctuation, and the energy of the binding value gradually decreases during dynamics after an initial increase (Figure S6). Compound **3** is the only one to show an average positive value with respect to the initial energy of the ligand–protein complex.

Analysis of dynamic cross-correlation matrices (DCCM) (Figure S7) showed that movements within domains, for the most part, are highly correlated, with few exceptions. The distal portion of the domain contains the active site has motions correlated with the rest of the domains. The root mean square fluctuation (RMSF) (Figure S8) confirms the good stability of the catalytic region with an important bending in the region involving the Leu200–Gly209 residues.

MD simulations for the BoNT/A light chain free state were performed to compare possible anomalous variations within the protein domains. The protein structure reaches a plateau after 30 ns, maintaining an almost constant RMSD for the remainder of the simulation (Figure S9).

A relatively higher RMSF value is obtained around the 60–80 residues, while optimal stability occurs in the remaining domains, especially in the region of the catalytic site. These data are confirmed by the good correlation between the different domains shown by the DCCM plot (Figure S9). A comparative analysis between the complex and free state of LC/A shows that the enzyme’s catalytic site is more prone to slight fluctuations when it forms complexes, despite all the complexes with the ligands showing positive correlations between the various domains.

Although our study is confined to the search of LC/A inhibitors, because the active site of LC of all serotypes is highly related, due to their high structural similarity, we also conducted in silico studies on BoNT LC catalytic domains of the B−G serotypes to investigate the inhibition specificity. For this purpose, the 10 most active LC/A inhibitors of Table 1 were docked into the Zn-dependent catalytic domain of LC/B−G proteases, considering the bidentate chelation of Zn^2+^ as the discriminating element. The results demonstrate a high selectivity of these compounds for the LC/A (Table S3), confirming the efficacy of our workflow for the discovery of novel BoNT LC/A inhibitors. Interestingly, compound ZINC5729284 showed a fair affinity towards all serotypes (Table S2), with a value of −8.7 kcal/mol for LC/B, probably due to the smaller size with respect to the other compounds, highlighting the possibility of developing broad-spectrum inhibitors against iatrogenic botulism. Most compounds (except **7**, **8**, and **10**) showed fair-to-good affinity against LC/G.

### 2.4. Workflow Model Validation and Molecular Diversity Assessment

To validate the entire procedure, we traced the CONTROL dataset (Table S1) down along the workflow path. The pharmacophore filter was passed by 10 of the 18 compounds (Table S1, light green background), six of which are the most powerful of all. These 10 compounds were also inserted in Table S2, highlighted with a light green background, to provide an immediate comparison of the number of CONTROL molecules that ended up in the list of the top best scorers. It is worth mentioning that the most potent predicted compound **1** (ZINC5008970) is in sixth place in Table S2, in the middle of the first seven ligands belonging to the CONTROL dataset. Successively, the IC_50_ and *K*_i_ activity values of the entire CONTROL dataset were calculated by employing the 3D-QSAR and docking protocols here presented, and were plotted versus the experimental ones (Table S1 and Figure S10). In both cases, we obtained an excellent correlation coefficient (*R*^2^ = 0.96 and 0.95 for the 3D-QSAR and docking methodologies, respectively; Figure S10), index of the excellent performance in predicting the values of the considered descriptors. Moreover, the replication of the pose of the crystallized ligand for the 2IMA X-ray structure (RMSD = 0.14 Å) by the alignment of the lower-energy pose obtained from molecular docking confirmed the accuracy of the docking algorithm (Figure S11).

Moreover, to more robustly assess the performance of the method used, we calculated the receiver operating characteristics (ROC) curves and the area under the ROC curves (ROC AUC), which are well-recognized metrics used as an objective way to evaluate the ability of a given test to discriminate between two populations [30], for the 3D-QSAR, docking, and mean approaches. As shown in Figure S12, all of the AUCs of the scoring functions are significantly larger than those of random distribution (the reference dashed line in Figure S12, AUC = 0.5), which indicates that the prediction capacity of these scoring functions is better than the random distribution model. Furthermore, the higher value of the AUC (0.884) obtained for the mean score function justifies using this consensus score as the best parameter for the ligands’ ranking. Similar results were obtained when the different models were evaluated for their early recognition capabilities by their enrichment factor (EF). EFs are more reliable towards the early recognition problem since they are focused on the true positive fraction. EFs express the number of active compounds found by employing a particular virtual screening strategy. The EF is a widely used validation tool for assessing the quality of virtual screening protocol [31]. The EF calculation results are represented in Figure S13 for the three models, showing maximum reachable EFs of 13.5 for both the 3D-QSAR and the consensus score parameter.

**Figure 6:**
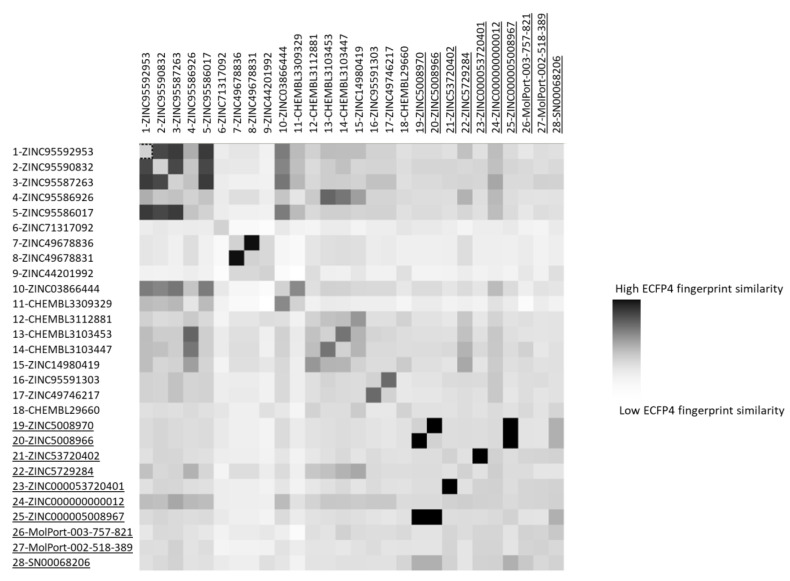
ECFP4 fingerprint similarity matrix.

Finally, to assess the molecular diversity of the compounds selected by our methodology, the pairwise similarity was calculated between the 18 LC/A binders of the CONTROL dataset and the 10 compounds reported in Table 1. The pairwise similarity was calculated by Forge using ECFP4/FCFP4 circular fingerprints [32]. The results are reported in Figure 6 and Figure S14. The results between classical LC/A ligands and our compounds are ranked at 0.017 and 0.347 using the ECFP4 fingerprint and between 0.045 and 0.457 when using the FCFP4 fingerprint. Both methodologies showed that the new set of selected compounds in Table 1 contain a wide range of molecularly different structures, from similar to very different compounds.

### 2.5. ADMET In Silico Properties

We carried out in silico ADMET studies on the ten molecules shown in Table 1 to highlight the results obtained from the 3D-QSAR and docking studies. Because an upstream selection of compounds with good ADMET properties could have ruled out potentially interesting, highly active inhibitors, we chose to perform the downstream ADMET computational evaluation, as it was previously proven to be effective [33,34].

The ability to reach targets in bioactive form was assessed using the SwissADME [35] web platform. It is important to highlight that the technologies exploited by this platform are able to predict, with a good approximation, the results generally observed in biochemical tests on small molecules [36]. Table 2 shows that seven compounds simultaneously satisfy Lipinski’s rule [37], and another four drug-likeness rules named Ghose [38], Egan [39], Veber [40], and Muegge [41]. In addition, seven out of ten molecules meet the lead-like criteria of Teague [42]. It is possible to deduce from the results obtained molecules **3**–**6** are excellent candidates for investigations based on the scaffold hopping approach. Finally, it is very important to underline that no molecule has shown alerts on the outcome of the PAINS model of the interference structures of the pan assay [43], which was designed to exclude small molecules that could show false positives in biological assays.

**Figure 7:**
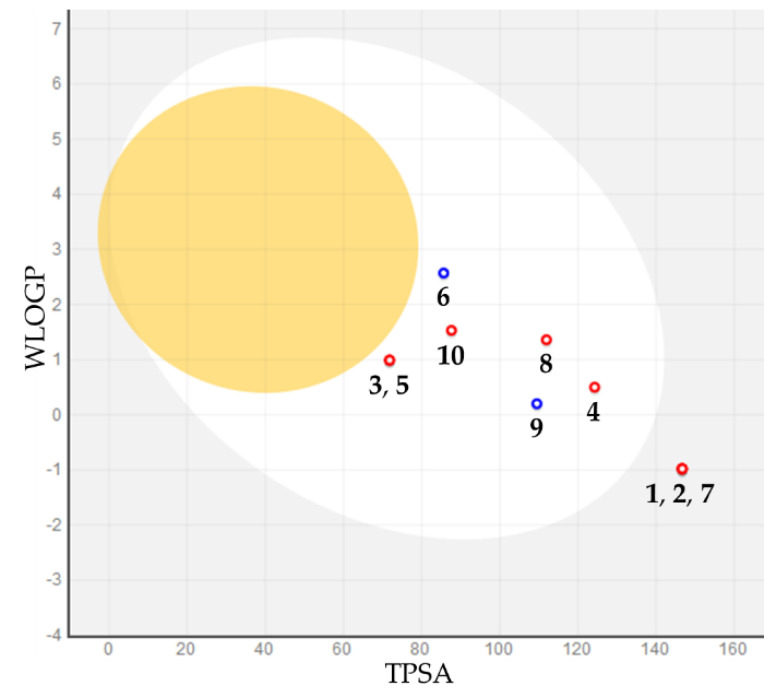
BOILED-Egg plot. Points located in the BOILED-Egg’s yolk (**yellow**) represent the molecules predicted to passively permeate through the blood–brain barrier, whereas those in the egg white are relative to the molecules predicted to be passively absorbed by the gastrointestinal tract; the blue dots indicate the molecules that were expected to be effluated from the central nervous system (CNS) by P-glycoprotein, whereas the red ones represent the molecules predicted not to be effluated from the CNS by P-glycoprotein.

Human gastrointestinal absorption (HIA) and blood–brain barrier penetration (BBB), relative to the absorption and distribution parameters, respectively, have been graphically represented by the extended and renewed version of the Edan–Egg model, named the brain or intestinal estimated (BOILED) permeation predictive model (BOILED-Egg, Figure 7). The results of the BOILED-Egg model overlap for molecules **3**, **5**, and **1**, **2**, and **7**. The visual analysis of Figure 7 highlights that all investigated molecules, with the exception of compounds **1**, **2**, and **7**, were predicted to be passively absorbed by the gastrointestinal tract, whereas none of them was predicted to permeate through the BBB passively. On the contrary, molecules **6** and **9** could be effluated from the central nervous system (CNS) with the aid of P-glycoprotein. These data are reflected in the values shown in Table 3.

Regarding the absorption parameters, all compounds present a poor-to-discrete oral availability due to the non-optimal Caco-2 cell permeability and HIA (should be >0.9 and >90%, respectively, Table 3), and a discrete skin permeability (the ideal value for a drug candidate for transdermal drug delivery is log*K*_p_ > −3.5, i.e., >0.5 cm/h, Table 3).

The volume of distribution (VD_ss_) and unbound fraction are two of the fundamental pharmacokinetic drug parameters. Values of the VD_ss_ > 0.45 indicate that the drug will be distributed in tissue, whereas values <−0.15 indicate that the drug will be distributed in plasma. Therefore, VD_ss_ describes the extent of drug distribution, and the unbound fraction describes the portion of free drug in plasma that may extravasate. The values obtained allow us to deduce that the molecules can be sufficiently distributed and have a significant fraction bound in the plasma that is available to interact with the drug target for a long time.

The total clearance values reported in Table 3, which assesses the body’s ability to eliminate a drug, show that all compounds, except molecule **4**, have good renal elimination (1.7–4.4 mL/min kg) and are not substrates of the renal organic cation transporter 2 (OCT2). Finally, all compounds, except **4**–**6** and **10**, passed all toxicity tests.

Table 3 underlines that almost all compounds could be suitable for valid candidates as drugs and could lead to further studies and manipulations. Moreover, compounds **1**, **2**, **4**–**9** possess all ideal properties of a drug candidate for transdermal drug delivery [45].

## 3. Materials and Methods

### 3.1. Dataset of Compounds

The chemical structures of the Marine Natural Product (MNP) and Super Natural Product (SNP) databases were retrieved from the website of Prof. Encinar [46] and uploaded to the Pharmit server, while the structures of the ZINC and Molport Natural (MPN) databases were directly available on the Pharmit Server. The CONTROL dataset list is reported in Table S1, whereas that of the 182 molecules filtered by the pharmacophore model (including those belonging to the CONTROL dataset) is available in Table S2.

### 3.2. Pharmacophore-Based Virtual Screening and Database Preparation

The pharmacophore model was generated using the Pharmit server [26], using the LC/A protease (PDB 2IMA) and 2,4-dichlorocinnamic hydroxamate ligand structures as input. Pharmit’s functions for 3D pharmacophore research have remained unchanged, except for the hydrophobic centers F4–6, whose radius has been set at 1.5 Å (Figure 1). This model was the basis for the virtual screening of the ZINC, SNP, MPN, and MNP libraries, which contained 13,592,287 molecules for a total of 126,606,488 conformers. Individual conformers with an RMSD ≥2 Å with respect to the ligand were discarded, while the remaining poses were minimized based on the functions of Pharmit.

### 3.3. Structure Preparation, Minimization, Molecular Docking, and Molecular Dynamics Simulations

The preparation of the structures of the molecules and the protein and all parameters for docking experiments and molecular dynamics simulations were conducted following the procedures previously reported by us [14,15,16,27,34]. The 3D structures of the BoNT LC/A−G (PDB ID: 4HEV, 1F82, 2QN0, 2FPQ, 1T3A, 2A8A, and 1ZB7) used for the experiments were obtained from the Protein Data Bank. The energy of binding along the MD simulation trajectories was calculated with the md_analyzebindenergy macro, implemented in the YASARA software, that accounts for the solvation effects.

In particular, flexible ligand docking experiments were performed by employing AutoDock 4.2.6 software implemented in YASARA (v. 19.5.5, YASARA Biosciences GmbH, Vienna, Austria) [29,47], using the Lamarckian genetic algorithm (LGA). The crystallized ligand was eliminated using YASARA software. The maps were generated by the program AutoGrid (4.2.6) with a spacing of 0.375 Å and dimensions that encompass all atoms extending 5 Å from the surface of the structure of the crystallized ligand. All parameters were inserted at their default settings, as previously reported [27]. In the docking tab, the macromolecule and ligand were selected, and GA parameters were set as ga_runs = 100, ga_pop_size = 150, ga_num_evals = 25,000,000, ga_num_generations = 27,000, ga_elitism = 1, ga_mutation_rate = 0.02, ga_crossover_rate = 0.8, ga_crossover_mode = two points, ga_cauchy_alpha = 0.0, ga_cauchy_beta = 1.0, and number of generations for picking worst individual = 10.

The molecular dynamics simulations were performed with the YASARA Structure package. A periodic simulation cell with boundaries extending 8 Å [48] from the surface of the complex was employed. The box was filled with water, with a maximum sum of all water bumps of 1.0 Å, and a density of 0.997 g mL^−1^.

The setup included optimizing the hydrogen bonding network 49] to increase the solute stability and a p*K*_a_ prediction to fine-tune the protonation states of protein residues at the chosen pH of 7.4 [49]. NaCl ions were added with a physiological concentration of 0.9%, with an excess of either Na or Cl to neutralize the cell. Water molecules were deleted to readjust the solvent density to 0.997 g/mL. The final system dimensions were 95 × 95 × 95 Å^3^ for each protein–ligand complex. All of the docking calculations generated 100 different conformations (binding poses). The best scored one in terms of predicted energy of binding (Δ*G*_B_) and zinc chelation (with very few exceptions, always the one with the lowest energy) was selected for each molecule.

The simulations were run using the ff14SB force field [50] for the solute, with GAFF2 [51] for non-standard residues, and TIP3P for water. The primary simulation was then run with a cutoff of 8 Å for van der Waals forces (the default used by AMBER) [52], and no cutoff was applied to electrostatic forces (using the particle mesh Ewald algorithm) [53]. The ligand force field parameters were generated with the AutoSMILES utility [54], which employs semiempirical AM1 geometry optimization and assignment of charges, followed by assignment of the AM1BCC [54] atom and bond types with refinement using the RESP charges, and finally, the assignments of general AMBER force field atom types. Optimization of the hydrogen bond network of the various enzyme–ligand complexes was obtained using the method established by Hooft et al. [55] to address ambiguities arising from multiple side-chain conformations and protonation states that are not well resolved in the electron density [56]. The entire system was then energy minimized using first a steepest descent minimization to remove conformational stress, followed by a simulated annealing minimization until convergence (<0.01 kcal/mol Å). The MD simulation was then initiated, using the NPT ensemble at 298 K and integration time steps for intramolecular and intermolecular forces every 1.25 and 2.5 fs, respectively.

Finally, 100 ns MD simulations without any restrictions were conducted, and the conformations of each system were recorded every 200 ps. On the averaged structure of the last 3 ns frames, a second cycle of energy minimization, identical to the first, was applied. After inspection of the solute RMSD as a function of simulation time, the last 3 ns-averaged structures were considered for further analysis.

### 3.4. Compound Alignment for the 3D-QSAR Study

A previously reported QSAR model was used to evaluate the 182 selected compounds by aligning them to the original model (made with a different set of selected molecules with the previously reported IC_50_) and evaluating their activity as follows: All of the fully PM3-optimized molecules were first imported into the software used for the 3D-QSAR calculation (Forge, v10.4.2, Cresset, New Cambridge House, Hertfordshire, UK). The field points were then calculated for each molecule; to this aim, the XED (extended electron distribution) force field was used. The conformational analysis was then conducted. For each molecule, a maximum of five hundred conformers were generated; 0.1 kcal/mol was used as a gradient cutoff for the conformer minimization, and 0.5 Å was used as a similarity threshold, below which two conformations were assumed identical. Then, 2.5 kcal/mol was set as an energy window for the conformations calculation; all other conformers with energy outside the energy window of 2.5 kcal/mol were discarded. Finally, all of the molecules were aligned to the original LC/A model [27] by a maximum common substructure algorithm using a customized and validated setup [57].

### 3.5. Building and Analysis of ROC Curves and EFs

For the evaluation and validation of the scoring functions, a test set covering active and inactive ligands was established by generating 438 ligands (the true negative, referred to as decoys) starting from the 18 ligands of the CONTROL panel (the true positive) employing the DUD-E webserver [58]. The decoy compounds have similar physicochemical properties but different 2D topology. The prepared active and decoy ligands were evaluated by the 3D-QSAR and docking methodologies, respectively. For docking, the top-scoring pose of each ligand, along with its docking score (Δ*G*_B_), was used for further analysis. All ranked lists were preprocessed in Microsoft Excel (Microsoft Inc., Albuquerque, NM, USA) and analyzed by the Screening Explorer tool available online [59].

### 3.6. In Silico ADMET Studies

In silico molecular studies were conducted with the use of SwissADME [35] and pkCSM [44] web platforms.

## 4. Conclusions

In this study, we highlight the importance of physicians knowing the antidotes for iatrogenic botulism when faced with cases of occasional adverse effects and overuse.

In a multidisciplinary group of neurologists, physiatrists, and chemists, we identified the clinical problem related to our outpatient treatments in treating spasticity by botulinum toxin in which it is possible to incur adverse events that we do not have an antidote for. In this paper, we applied a well-defined method based on ligand and structure through an initial pharmacophore filter and a previously developed 3D-QSAR model. Unlike the pharmacophore models developed in the past, which did not succeed in finding compounds with *K*_i_ > 300, we chose to use the most potent known crystallized inhibitor (DCNHA) with the aim of identifying small molecules with potential activity in the low nM. The sub-linear algorithms of the Pharmit server, already used in a previous study, allowed us to reduce the initial molecules from 13,592,287 to 172 through an initial virtual screening based on pharmacophore support. The average values of the pIC_50_ and p*K*_i_ obtained from the 3D-QSAR model in addition to accurate docking studies allowed for the identification of the substances with higher predicted affinity with the LC/A binding pocket. The entire workflow model was validated employing a CONTROL dataset, and the molecular diversity of the predicted most potent compounds with respect to the CONTROL inhibitors was assessed by pairwise similarity analysis. Furthermore, the ROC curves and the AUCs values were calculated for the better reliability of the method used.

Although the most active substances are compounds obtained from synthesis (commercially available), other potential LC/A inhibitors identified in this study are of natural origin. In fact, compounds isolated from plants can represent a potential low-cost drug treatment [60]. All the structures selected for MD simulation chelated the Zn^2+^ ion with bidentate coordination inside the binding pocket, maintaining the bond throughout the simulation. This is confirmed by the DCCM and RMSF analysis, demonstrating the good stability of the catalytic domain in the protein–ligand complex. The re-docking approach also assessed the good stability of the protein–ligand complex. The poses of compounds **3** and **4** suggest that the sub-pocket, which includes His223 and Arg363 residues, may be used to optimize the drug design for future BoNT/A LC inhibitors.

Docking studies across all BoNT LC serotypes have shown that the top 10 LC/A inhibitors have high selectivity, highlighting that small inhibitors (such as ZINC5729284) could be extremely valuable due to their inhibitory activity for the treatment of botulism.

## Figures and Tables

**Table 1:** 3D-QSAR: calculated free energies of binding by docking (Δ*G*_B_, in kcal/mol), p*K*_i_ (docking structure-based) values, mean results (a consensus score as the average of the 3D-QSAR pIC_50_ and docking p*K*_i_ values), and re-docking for the compounds with the best score.

Compound/ID	Structure	3D-QSAR pIC_50_	Δ*G*_B_	Docking p*K*_i_	Mean	Re-Docking p*K*_i_
**1** ZINC5008970	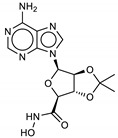	6.3	−9.2	6.8	6.5	6.6
**2** ZINC5008966	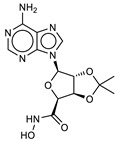	6.1	−8.9	6.5	6.3	6.4
**3** ZINC53720402	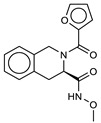	5.9	−9.2	6.7	6.3	6.3
**4** ZINC5729284	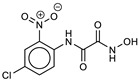	5.8	−9.2	6.7	6.2	6.5
**5** ZINC000053720401	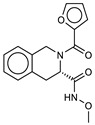	5.8	−9.1	6.7	6.2	—
**6** ZINC000000000012	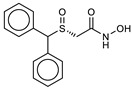	6.1	−8.7	6.4	6.2	—
**7** ZINC000005008967	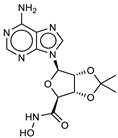	5.9	−8.9	6.5	6.2	—
**8** MolPort-003-757-821	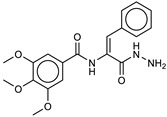	6.1	−8.6	6.3	6.2	—
**9** MolPort-002-518-389	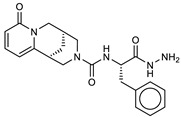	5.8	−9.0	6.6	6.2	—
**10** SN00068206	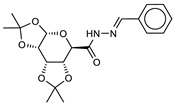	6.7	−9.1	5.7	6.2	—

**Table 2:** Drug-likeness, lead-likeness, and PAINS parameters of compounds reported in Table 1 ^a^.

Compound		1	2	3	4	5	6	7	8	9	10
Drug-likeness	Lipinski violations	1	1	0	0	0	0	1	0	0	0
	Ghose violations	1	1	0	0	0	0	1	0	0	0
	Veber violations	1	1	0	0	0	0	1	0	0	0
	Egan violations	1	1	0	0	0	0	1	0	0	0
	Muegge violations	0	0	0	0	0	0	0	0	0	0
Lead-likeness violations		0	0	0	0	0	0	0	2	1	1
PAINS alerts		0	0	0	0	0	0	0	0	0	0

^a^ All results were obtained from the SwissADMET webserver [35].

**Table 3:** Pharmacokinetic- and toxicity-evaluated parameters of compounds reported in Table 1 ^a,b^.

Compound		1	2	3	4	5	6	7	8	9	10
Absorption	Caco-2 permeability	−0.229	−0.229	0.738	−0.136	0.892	0.833	0.226	−0.193	0.244	0.781
	Human intestinal absorption	72.208	72.208	95.203	72.88	94.924	94.829	80.162	65.507	75.034	95.909
	Skin permeability	−2.735	−2.735	−3.318	−2.855	−2827	−2.777	−2.735	−2.915	−2.763	−3.461
Distribution	VD_ss_ (human)	0.444	0.444	−0.165	−1.026	0.129	−0.283	0.213	−0.386	−0.008	−0.147
	Fraction unbound (human)	0.736	0.736	0.335	0.425	0.408	0.094	0.705	0.151	0.367	0.323
	BBB permeability	−1.378	−1.378	−0.208	−1.425	−0.279	−0.172	−1.334	−0.941	−0.734	−0.67
	CNS permeability	−3.874	−3.874	−2.518	−2.913	−2.447	−2.607	−3.363	−3.062	−2.963	−3.207
Excretion	Total clearance	0.644	0.644	0.515	−0.058	0.498	0.582	0.647	0.226	0.552	1.141
	Renal OCT2 substrate ^c^	No	No	No	No	No	No	No	No	No	No
Toxicity	Skin sensitization	No	No	No	No	No	No	No	No	No	No
	AMES toxicity	No	No	No	Yes	Yes	Yes	No	No	No	Yes
	Oral rat acute toxicity (LD50)	2.275	2.275	0.874	2.984	2.368	2.512	2.401	2.852	2.862	2.774
	Minnow toxicity	3.169	3.169	2.94	1.987	2.437	−0.023	4.045	−0.275	2.108	−0.42

^a^ All results were obtained from the pkCSM webserver [44]. ^b^ Semaphore flags: green = excellent, yellow = discrete, red = insufficient. ^c^ Unimportant, because the total clearance is high, with the exception of compound **4**.

## Supplementary Materials

Supplementary materials can be found at https://www.mdpi.com/1422-0067/21/24/9470/s1.
