# Supplementary material for: An Integrated Pharmacophore/Docking/3D-QSAR Approach to Screening a Large Library of Products in Search of Future Botulinum Neurotoxin A Inhibitors
Source: Int J Mol Sci. 2020 Dec 12;21(24):9470. doi: 10.3390/ijms21249470 (PMC7764241; doi:10.3390/ijms21249470)
Supplement: Supplementary file 1 [file ijms-21-09470-s001.pdf]

International Journal of
Molecular Sciences

MDPI

Supplementary Materials

An Integrated Pharmacophore/Docking/3D-QSAR Approach to Screening a Large Library of Products in Search of Future Botulinum Neurotoxin A Inhibitors

Davide Gentile [1], Giuseppe Floresta [1], Vincenzo Patamia [1], Rita Chiaramonte [2], Giulia Letizia Mauro [3], Antonio Rescifina [1,*] and Michele Vecchio [2,*]

[1] Department of Drug Sciences, University of Catania, V.le A. Doria, 95125 Catania, Italy, davide.gentile@unict.it (D.G.), giuseppe.floresta@unict.it (G.F.), vincenzo.patamia@unict.it (V.P.), arescifina@unict.it (A.R.)

[2] Department of Biomedical and Biotechnological Sciences, Section of Pharmacology, University of Catania, Via S. Sofia 67, 95123 Catania, Italy, michele.vecchio@unict.it (M.V.), ritachiaramd@gmail.com (R.C.)

[3] Department of Surgery, Oncology, and Stomatology, University of Palermo, Via Liborio Giuffrè 5, 90127 Palermo, Italy, giulia.letiziamauro@unipa.it (G.L.M.)

* Correspondence: arescifina@unict.it; Tel.: +39-095-738-5017 (A.R.), michele.vecchio@unict.it (M.V.)

Table of content

**Table S1.**	ID, experimental and predicted K_i and IC_{50} values (nM), and means of the calculated values (expressed as –log of the mean value) of selected LC/A inhibitors used as CONTROL to validate the workflow performance.	S3
**Table S2.**	ID of compounds that passed the pharmacophore filter, including predicted pIC_{50} (3D-QSAR ligand-based), calculated free energies of binding by docking (ΔG_B, in kcal/mol), pK_i (Docking structure-based) values, and their mean.	S3
**Figure S1.**	Docking binding pose (up-left) and interaction (up-right) inside the binding pocket of the protein. Total energy (down-left) and RMSDs (downright) of protein and its complexes with ZINC5008970 ligand.	S7
**Figure S2.**	Docking binding pose (up-left) and interaction (up-right) inside the binding pocket of the protein. Total energy (down-left) and RMSDs (downright) of protein and its complexes with ZINC5008966 ligand.	S8
**Figure S3.**	Docking binding pose (up-left) and interaction (up-right) inside the binding pocket of the protein. Total energy (down-left) and RMSDs (downright) of protein and its complexes with ZINC53720402 ligand.	S8
**Figure S4.**	Docking binding pose (up-left) and interaction (up-right) inside the binding pocket of the protein. Total energy (down-left) and RMSDs (downright) of protein and its complexes with ZINC5729284 ligand.	S9
**Figure S5.**	Distance variation of the hydroxyl H-bond during the MD simulation of compounds **1**, **2**, and **4** with the residue Glu224.	S9
**Figure S6.**	Variation of the energy of binding along the MD simulation trajectory of compounds **1**–**4**.	S10
**Figure S7.**	Dynamic cross-correlation matrix of compounds **1**–**4**. The DCCM is visualized with colors ranging from blue (−1, fully anti-correlated) to yellow (+1, fully correlated).	S10
**Figure S8.**	The Root Mean Square Fluctuation (RMSF) [vertical axis] per solute protein residue [horizontal axis] calculated from the average RMSF of the atoms constituting the residue of compounds **1**–**4**.	S11
**Figure S9.**	Total energy (up-left), DCCM (up-right), RMSF (down-left) and RMSD (down-right) of BoNT/A Light Chain free state.	S11
**Table S3.**	Structures and calculated free energies of binding (ΔG_B, in kcal/mol) of the selected compounds **1**–**10** for the LC serotypes B, C, D, E, F, and G.	S12

Figure S10. Linear regression plots and correlation coefficients of calculated *vs*. experimental IC_{50} (left) and K_i (right) values obtained for CONTROL dataset. Values from Table S1. S12
Figure S11. Crystallized laying (yellow) and docked pose (green) of (2*E*)-3-(2,4-dichlorophenyl)-*N*-hydroxyacrylamide inside the binding pocket with the respective values of root mean square (RMSD) and experimental and predicted K_i values. S13
Figure S12. ROC curves for the scoring functions. S13
Figure S13. Enrichment curves for the scoring functions. S14
Figure S14. FCFP4 fingerprint similarity matrix. S14

Table S1. ID, experimental and predicted K_i and IC_{50} values (nM), and means of the calculated values (expressed as –log of the mean value) of selected LC/A inhibitors used as CONTROL dataset to validate the workflow performance [a].

CONTROL dataset	3D-QSAR			Docking		Mean
ID of LC/A inhibitor	Exp. IC_{50}	Calcd. IC_{50}	Distance to model	Exp. K_i (nM)	Calcd. K_i	–log
ZINC95592953	30	79	Excellent	27	36	7.2
ZINC95586017	40	79	Excellent	27	37	7.2
CHEMBL3112881	150	180	Excellent	77	147	6.8
ZINC95590832	250	199	Excellent	130	110	6.8
ZINC95586926	— [b]	274	Excellent	160	149	6.7
ZINC14980419	410	450	Excellent	300	320	6.4
CHEMBL3309329	1500	1584	Excellent	460	450	6.0
ZINC03866444	1000	1000	Excellent	460	480	6.1
ZINC49678831	—	N.D. [c]		600	590	
ZINC49678836		N.D.		600	630	
ZINC95587263	500	316	Poor	750	428	6.4
ZINC95591303	—	N.D.		760	810	
ZINC49746217	—	N.D.		760	746	
ZINC71317092	1500	1356	Bad	800	923	5.9
CHEMBL3103447	1100	1258	Excellent	1000	991	5.9
CHEMBL3103453	970	794	Poor	2100	1527	5.9
CHEMBL29660	—	N.D.		1400	1337	
ZINC44201992	—	N.D.		3000	3128	

[a] The compounds highlighted with a light green background are those that have passed the pharmacophore filter. [b] The value is not present in literature. [c] N.D. = not done.

Table S2. ID of compounds that passed the pharmacophore filter, including predicted pIC_{50} (3D-QSAR ligand-based), calculated free energies of binding by docking (ΔG_B, in kcal/mol), pK_i (Docking structure-based) values, and their mean [a].

ID Compound	Distance to model	3D-QSAR results pIC_{50}	ΔG_B	Docking results pK_i	Mean results
ZINC95592953	Excellent	7.1	−10.1	7.4	7.2
ZINC95586017	Excellent	7.1	−10.1	7.4	7.2
CHEMBL3112881	Excellent	6.7	−9.3	6.8	6.8
ZINC95590832	Excellent	6.7	−9.5	7.0	6.8
ZINC95586926	Excellent	6.6	−9.3	6.8	6.7
ZINC000005008970	OK	6.3	−9.2	6.7	6.5
ZINC14980419	Excellent	6.3	−8.8	6.5	6.4
ZINC95587263	Poor	6.5	−8.7	6.4	6.4
ZINC000005008966	Excellent	6.3	−8.9	6.5	6.4
ZINC000053720402	Good	5.9	−9.2	6.7	6.3
ZINC000005729284	Excellent	5.8	−9.2	6.7	6.3
MolPort-042-671-961	Bad	6	−8.9	6.5	6.3
ZINC000053720401	Good	5.8	−9.1	6.7	6.2
ZINC000000000012	OK	6.1	−8.7	6.4	6.2
ZINC000005008967	Good	5.9	−8.9	6.5	6.2

MolPort-003-757-821	Poor	6.1	−8.6	6.3	6.2
MolPort-002-518-389	OK	5.8	−9.0	6.6	6.2
SN00068206	OK	5.7	−9.1	6.7	6.2
ZINC03866444	Excellent	6.0	−8.6	6.3	6.1
MolPort-002-665-387	OK	5.2	−9.5	7.0	6.1
SN00427215	Poor	5.0	−9.9	7.3	6.1
SN00046401	Good	5.9	−8.6	6.3	6.1
MolPort-044-180-385	Poor	5.6	−9.0	6.6	6.1
MolPort-027-564-680	Poor	5.6	−9.0	6.6	6.1
SN00070021	Poor	5.4	−9.3	6.8	6.1
MolPort-039-057-028	Bad	4.9	−9.9	7.3	6.1
SN00068180	Poor	5.8	−8.9	6.5	6.1
MolPort-000-740-854	OK	5.0	−9.8	7.2	6.1
MolPort-000-856-378	Bad	4.9	−9.9	7.2	6.1
MolPort-027-718-044	OK	5.8	−8.6	6.3	6.1
MolPort-002-667-108	OK	5.2	−9.4	6.9	6.0
MolPort-039-347-777	Excellent	5.4	−9.0	6.6	6.0
CHEMBL3103447	Excellent	5.9	−8.2	6.0	5.9
CHEMBL3103453	Poor	6.1	−7.9	5.8	5.9
SN00320933	Excellent	6.0	−8.0	5.9	5.9
MolPort-005-946-143	Bad	5.1	−9.3	6.8	5.9
MolPort-002-518-329	Good	5.7	−8.4	6.1	5.9
MolPort-002-515-987	Bad	5.6	−8.5	6.2	5.9
MolPort-000-854-636	Bad	5.2	−9.0	6.6	5.9
MolPort-028-854-037	Bad	4.8	−9.5	7.0	5.9
SN00124946	Good	5.5	−8.6	6.3	5.9
MolPort-000-786-596	Poor	5.2	−8.9	6.5	5.9
MolPort-044-543-795	Good	4.7	−9.6	7.0	5.9
MolPort-002-536-306	Bad	5.2	−8.9	6.5	5.8
SN00124947	Excellent	5.4	−8.6	6.3	5.8
MolPort-002-513-193	Poor	5.9	−7.8	5.7	5.8
SN00375125	Poor	5.4	−8.3	6.1	5.8
MolPort-002-518-279	OK	4.6	−9.4	6.9	5.8
MolPort-028-854-620	OK	5.3	−8.4	6.2	5.7
MolPort-002-526-116	Poor	4.6	−9.4	6.9	5.7
ZINC000134769536	Excellent	5.4	−8.3	6.1	5.7
SN00136907	Bad	5.8	−7.7	5.7	5.7
MolPort-000-844-026	OK	4.9	−8.9	6.5	5.7
SN00127503	Good	4.9	−8.9	6.5	5.7
ZINC000004362771	Excellent	4.9	−8.8	6.5	5.7
MolPort-002-534-728	Poor	5.7	−7.7	5.6	5.7
MolPort-002-665-378	OK	4.7	−9.0	6.6	5.7
SN00006627	Excellent	5.4	−8.1	5.9	5.7
MolPort-002-535-110	Poor	5.4	−8.1	5.9	5.7
SN00099422	Good	5.4	−8.1	5.9	5.7
SN00017939	Excellent	5.7	−7.6	5.6	5.6
MolPort-000-727-325	Poor	5.1	−8.4	6.2	5.6
MolPort-039-056-168	OK	5	−8.5	6.2	5.6
MolPort-009-759-098	Poor	5.3	−8.0	5.9	5.6
MolPort-005-916-122	Poor	4.8	−8.7	6.4	5.6
SN00083645	Bad	5.1	−8.3	6.1	5.6

SN00141209	OK	5.1	−8.2	6.0	5.6
MolPort-005-912-756	OK	5	−8.4	6.1	5.6
MolPort-002-535-357	OK	4.9	−8.5	6.2	5.6
MolPort-001-943-322	OK	4.8	−8.6	6.3	5.6
MolPort-019-825-766	Poor	4.7	−8.7	6.4	5.6
ZINC000226290837	Good	5.2	−8.0	5.9	5.5
MolPort-000-853-362	OK	5.5	−7.5	5.5	5.5
MolPort-002-535-836	Bad	4.7	−8.6	6.3	5.5
SN00111822	Poor	5.5	−7.5	5.5	5.5
ZINC000134764989	Excellent	5.6	−7.3	5.4	5.5
MolPort-005-911-023	Good	4.7	−8.5	6.2	5.5
MolPort-000-740-476	Bad	5.3	−7.7	5.6	5.5
ZINC000003907070	Excellent	5.1	−7.9	5.8	5.5
ZINC000134769485	Excellent	5.6	−7.2	5.3	5.5
MolPort-007-740-139	Excellent	5.8	−7.0	5.1	5.4
MolPort-002-686-968	Poor	5	−8.0	5.9	5.4
SN00045599	OK	5.8	−6.9	5.1	5.4
SN00142379	Good	5.4	−7.4	5.5	5.4
ZINC000585136822	OK	5.4	−7.4	5.5	5.4
ZINC000217595499	Excellent	5.3	−7.6	5.6	5.4
ZINC000063727176	Excellent	5.2	−7.7	5.6	5.4
MolPort-003-959-819	Bad	5.5	−7.2	5.3	5.4
ZINC000036391296	Excellent	5.5	−7.2	5.3	5.4
MolPort-009-649-390	Bad	4.8	−8.2	6.0	5.4
SN00000073	Good	5.4	−7.4	5.4	5.4
ZINC000217595402	Excellent	5.4	−7.2	5.3	5.4
MolPort-000-741-314	Poor	6	−6.4	4.7	5.4
MolPort-028-855-574	Bad	4.5	−8.5	6.2	5.3
ZINC000036391297	Excellent	5.5	−7.0	5.1	5.3
MolPort-028-853-229	Poor	4.7	−8.1	5.9	5.3
ZINC000134758738	Excellent	5.2	−7.4	5.4	5.3
ZINC000005497494	Excellent	4.7	−8.0	5.9	5.3
MolPort-002-515-291	OK	5.3	−7.2	5.3	5.3
ZINC000585136821	Excellent	5.2	−7.3	5.4	5.3
ZINC000134753043	OK	5.2	−7.3	5.4	5.3
ZINC000134764032	Good	5.3	−7.2	5.3	5.3
SN00128684	Bad	4.4	−8.4	6.2	5.3
MolPort-002-669-519	Excellent	4.7	−8.0	5.9	5.3
MolPort-035-873-215	Bad	4.6	−8.1	5.9	5.3
SN00064762	Excellent	5.5	−6.9	5.0	5.3
ZINC000068135080	Excellent	4.6	−8.1	5.9	5.3
MolPort-002-515-774	Poor	4.9	−7.7	5.6	5.3
SN00001100	Good	5.6	−6.7	4.9	5.2
ZINC000217595584	OK	4.8	−7.8	5.7	5.2
ZINC000134771067	Excellent	5.1	−7.3	5.4	5.2
ZINC000134745254	OK	4.9	−7.6	5.5	5.2
MolPort-002-509-196	Excellent	4.9	−7.6	5.5	5.2
ZINC000134766123	Good	5.2	−7.1	5.2	5.2
ZINC000036391298	Excellent	5.2	−7.1	5.2	5.2
MolPort-002-515-700	OK	5.0	−7.4	5.4	5.2
ZINC000167074621	Excellent	4.6	−7.9	5.8	5.2

ZINC000065491095	Excellent	5.2	−7.1	5.2	5.2
SN00137214	Good	5.2	−7.1	5.2	5.2
ZINC000134755053	Excellent	5.5	−6.7	4.9	5.2
ZINC000134771652	OK	5.2	−7.0	5.2	5.2
MolPort-000-400-189	Excellent	4.8	−7.6	5.6	5.2
ZINC000010278186	Excellent	4.7	−7.7	5.7	5.2
SN00045598	Excellent	5.9	−6.1	4.5	5.2
ZINC000032848322	Excellent	5.4	−6.7	4.9	5.2
MolPort-001-930-277	Good	4.7	−7.7	5.6	5.2
ZINC000056213930	Excellent	4.8	−7.5	5.5	5.2
MolPort-002-514-114	Poor	5.7	−6.3	4.6	5.2
ZINC000134755144	Excellent	4.9	−7.4	5.4	5.1
SN00117433	Good	5.4	−6.7	4.9	5.1
ZINC000058279590	Excellent	4.8	−7.5	5.5	5.1
ZINC000134764762	Excellent	4.6	−7.8	5.7	5.1
ZINC000058477010	Excellent	5.3	−6.8	5.0	5.1
SN00215130	Excellent	5.5	−6.5	4.8	5.1
ZINC000134754140	Excellent	4.8	−7.4	5.5	5.1
ZINC000217596838	Poor	4.9	−7.3	5.3	5.1
ZINC000028099433	Good	4.6	−7.6	5.6	5.1
SN00139212	Excellent	5.7	−6.1	4.4	5.1
ZINC000012583748	Excellent	4.4	−7.8	5.7	5.1
ZINC000036391295	Excellent	4.7	−7.4	5.4	5.1
ZINC000082770562	Excellent	4.8	−7.3	5.3	5.1
SN00136991	OK	4.9	−7.1	5.2	5.1
MolPort-002-507-981	Excellent	5.1	−6.8	5.0	5.0
SN00131765	Poor	5.6	−6.1	4.5	5.0
ZINC000134739255	Good	4.6	−7.4	5.4	5.0
ZINC000134762404	Excellent	4.8	−7.1	5.2	5.0
ZINC000019436641	OK	4.6	−7.4	5.4	5.0
SN00035144	Good	4.9	−6.9	5.1	5.0
SN00101969	Excellent	5.3	−6.4	4.7	5.0
ZINC000217596720	Excellent	5.0	−6.7	4.9	5.0
SN00120543	Poor	5.5	−6.0	4.4	5.0
SN00130258	Excellent	5.1	−6.5	4.8	4.9
SN00139213	Excellent	5.4	−6.1	4.5	4.9
ZINC000134762647	Excellent	4.9	−6.8	5.0	4.9
SN00139215	Excellent	5.4	−6.1	4.5	4.9
SN00101968	Excellent	4.9	−6.7	4.9	4.9
SN00131000	Excellent	5.4	−6.0	4.4	4.9
ZINC000005497599	Excellent	5.0	−6.6	4.8	4.9
SN00049824	Poor	4.9	−6.7	4.9	4.9
SN00131001	Excellent	5.5	−5.9	4.3	4.9
SN00139214	Excellent	5.5	−5.9	4.3	4.9
ZINC000032848242	Excellent	4.9	−6.7	4.9	4.9
ZINC000048311381	Good	4.6	−7.1	5.2	4.9
SN00035143	Excellent	5.0	−6.4	4.7	4.8
ZINC000134769510	Poor	4.8	−6.7	4.9	4.8
MolPort-001-562-279	Bad	4.9	−6.5	4.8	4.8
SN00120542	Excellent	5.2	−6.0	4.4	4.8
ZINC000082769613	Excellent	4.5	−6.9	5.1	4.8

SN00132813	Excellent	4.9	−6.3	4.6	4.8
MolPort-000-156-429	Excellent	4.5	−6.7	4.9	4.7
SN00131552	Poor	5.1	−5.9	4.3	4.7
MolPort-002-515-032	Excellent	4.9	−6.1	4.5	4.7
ZINC000017821388	OK	4.1	−7.1	5.2	4.7
ZINC000005997981	Good	4.3	−6.8	5.0	4.7
ZINC000065538427	Excellent	4.4	−6.6	4.8	4.6
MolPort-002-710-157	Excellent	4.7	−6.0	4.4	4.6
SN00141724	Poor	4.7	−6.0	4.4	4.5
SN00126424	Excellent	4.7	−5.8	4.3	4.5
SN00131487	Excellent	4.9	−5.5	4.1	4.5
ZINC000000500969	Good	4.2	−6.4	4.7	4.5
MolPort-001-917-139	Good	4.5	−5.6	4.1	4.3

[a] The compounds highlighted with a light green background are those that belong to the CONTROL dataset.

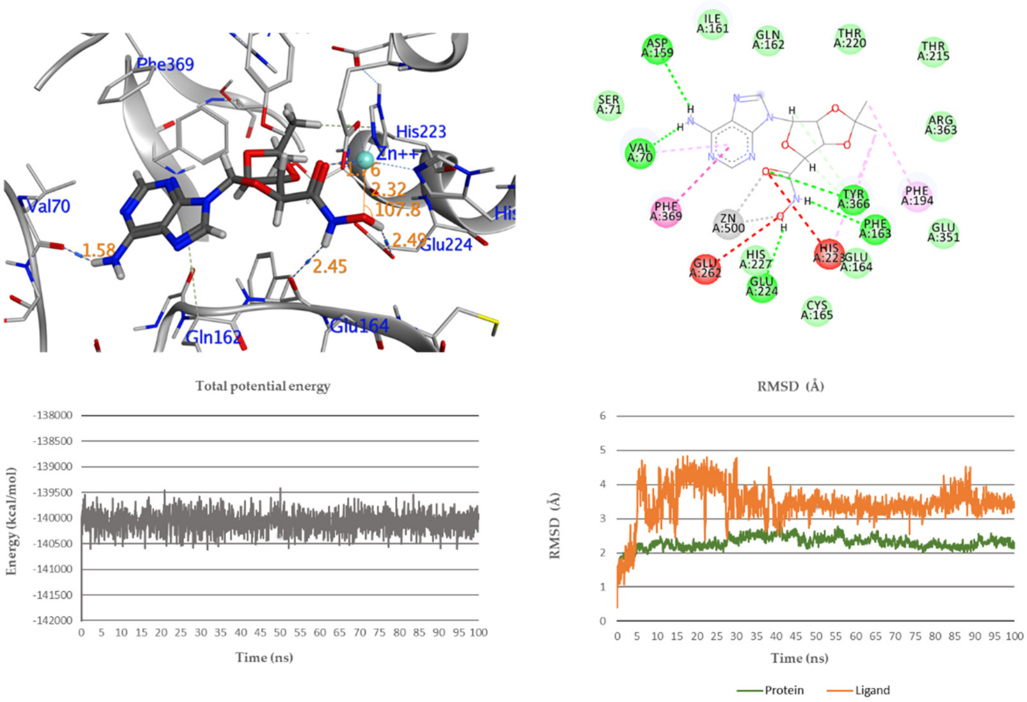

Figure S1. Docking binding pose (up-left) and interaction (up-right) inside the binding pocket of the protein. Total energy (down-left) and RMSDs (downright) of protein and its complexes with ligand ZINC5008970.

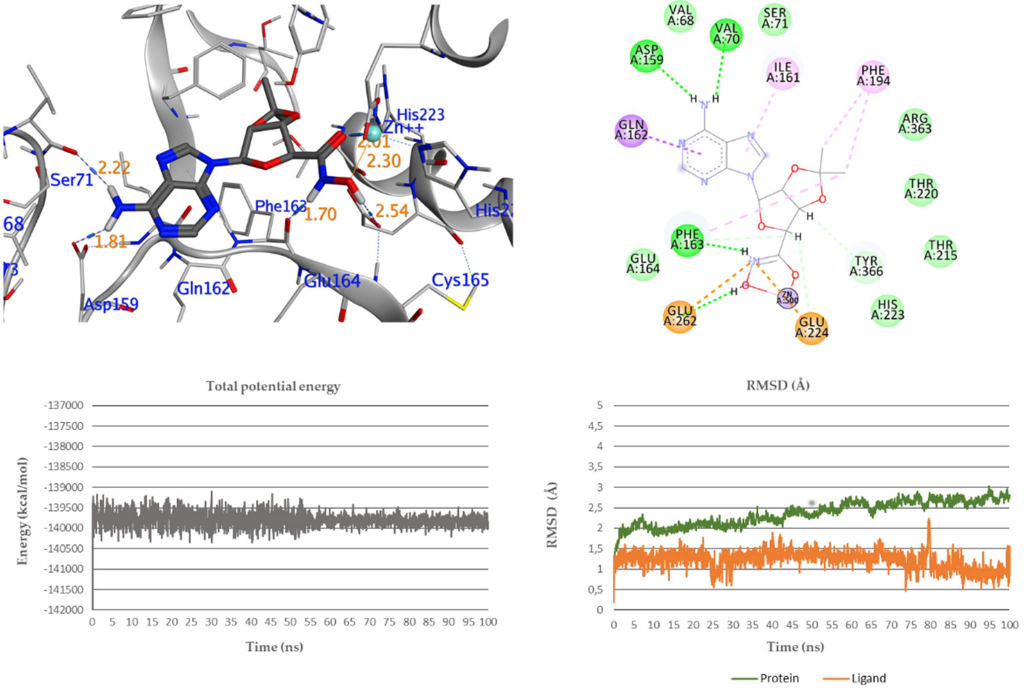

Figure S2. Docking binding pose (up-left) and interaction (up-right) inside the binding pocket of the protein. Total energy (down-left) and RMSDs (downright) of protein and its complexes with ligand ZINC5008966.

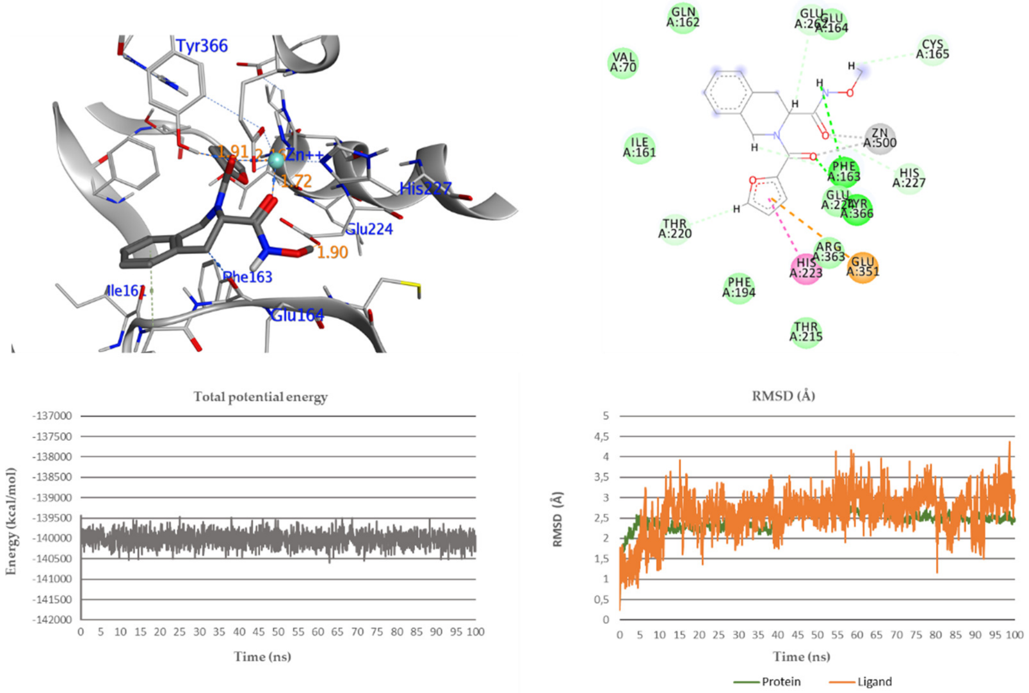

Figure S3. Docking binding pose (up-left) and interaction (up-right) inside the binding pocket of the protein. Total energy (down-left) and RMSDs (downright) of protein and its complexes with ligand ZINC53720402.

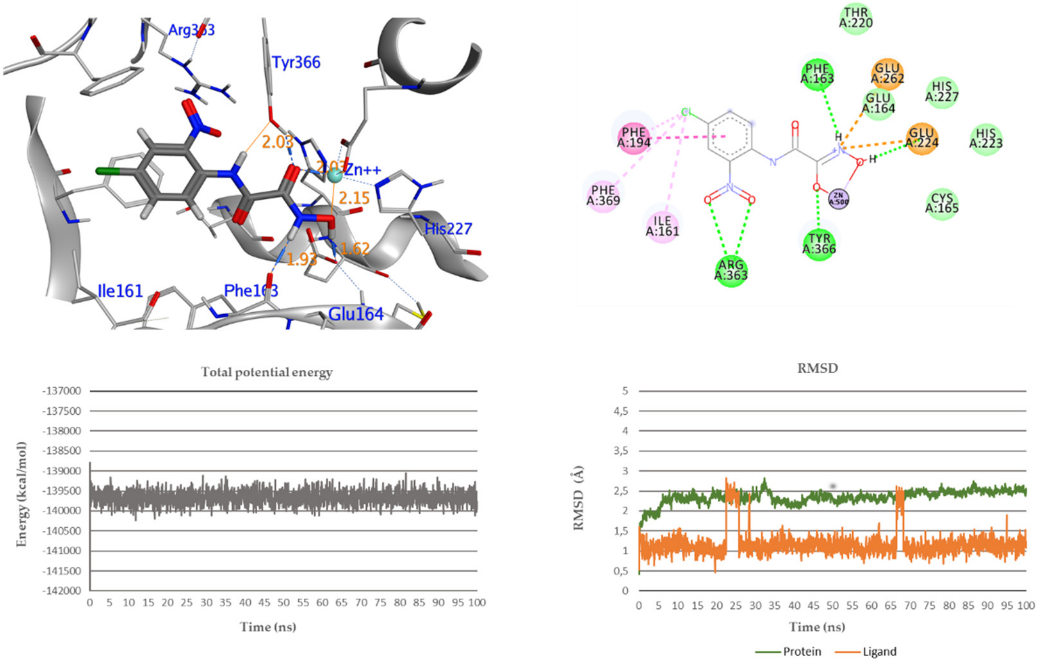

Figure S4. Docking binding pose (up-left) and interaction (up-right) inside the binding pocket of the protein. Total energy (down-left) and RMSDs (downright) of protein and its complexes with ZINC5729284 ligand.

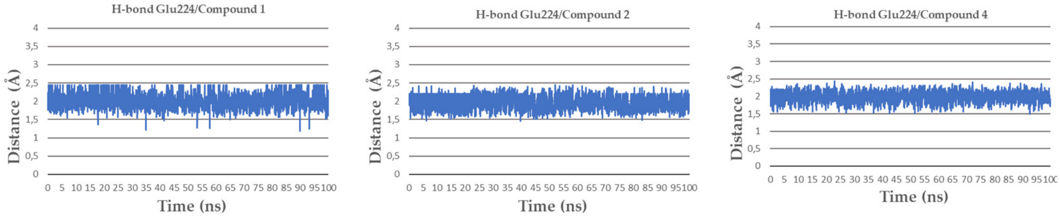

Figure S5. Distance variation of the hydroxyl H-bond during the MD simulation of compounds **1**, **2**, and **4** with the residue Glu224.

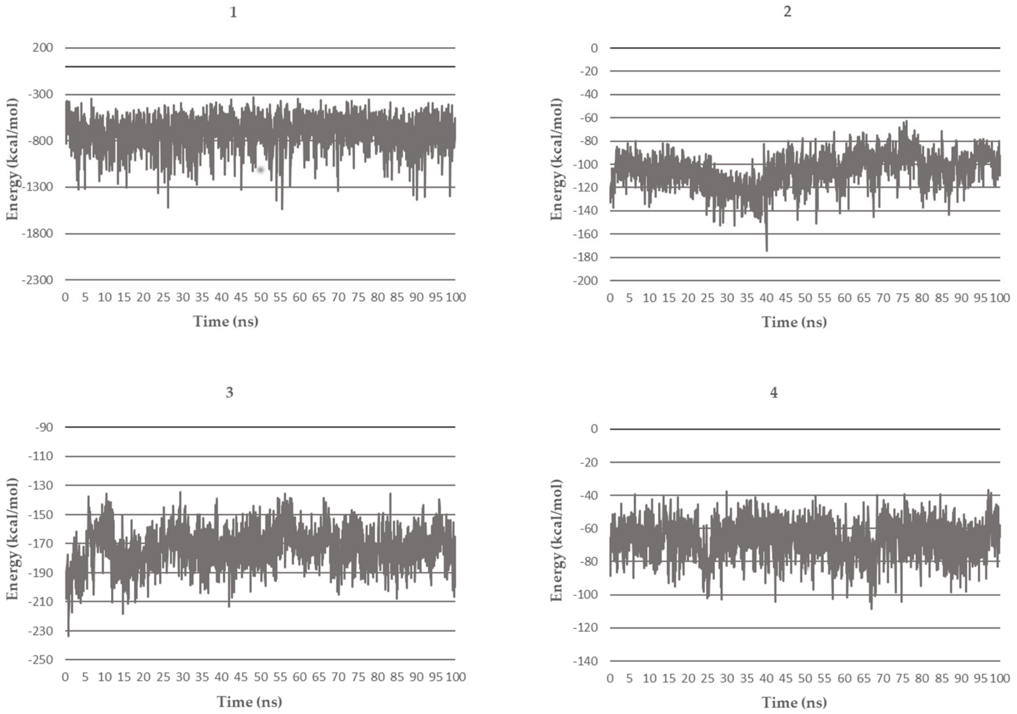

Figure S6. Variation of the energy of binding along the MD simulation trajectory for compounds **1**–**4**.

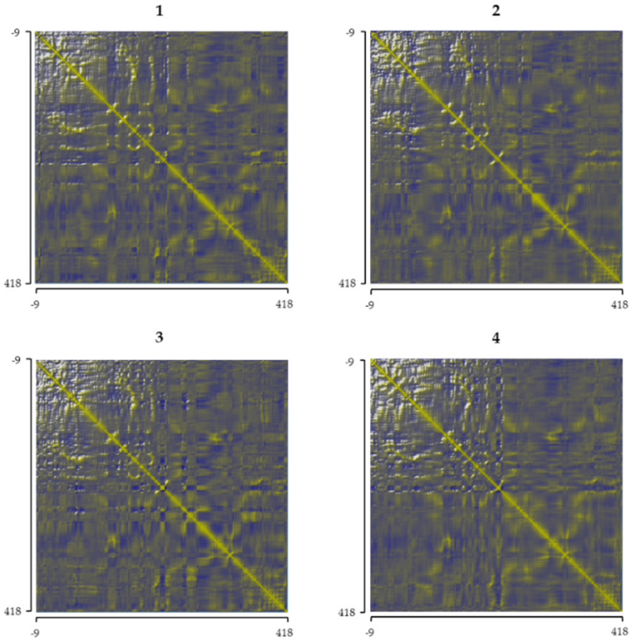

Figure S7. Dynamic cross-correlation matrix of compounds **1**–**4**. The DCCM is visualized with colors ranging from blue (−1, fully anti-correlated) to yellow (+1, fully correlated).

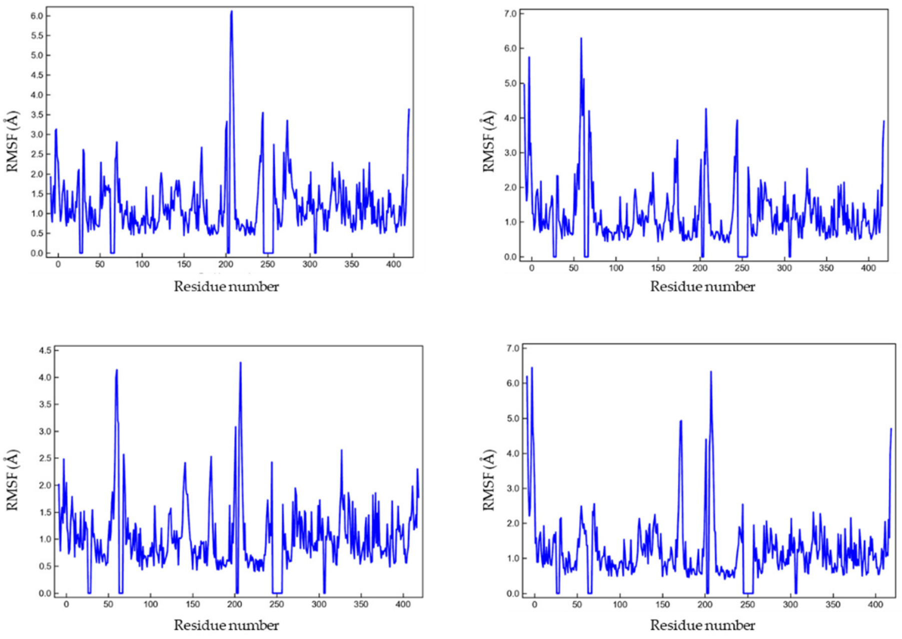

Figure S8. The Root Mean Square Fluctuation (RMSF) [vertical axis] per solute protein residue [horizontal axis] calculated from the average RMSF of the atoms constituting the residue of compounds **1**–**4**.

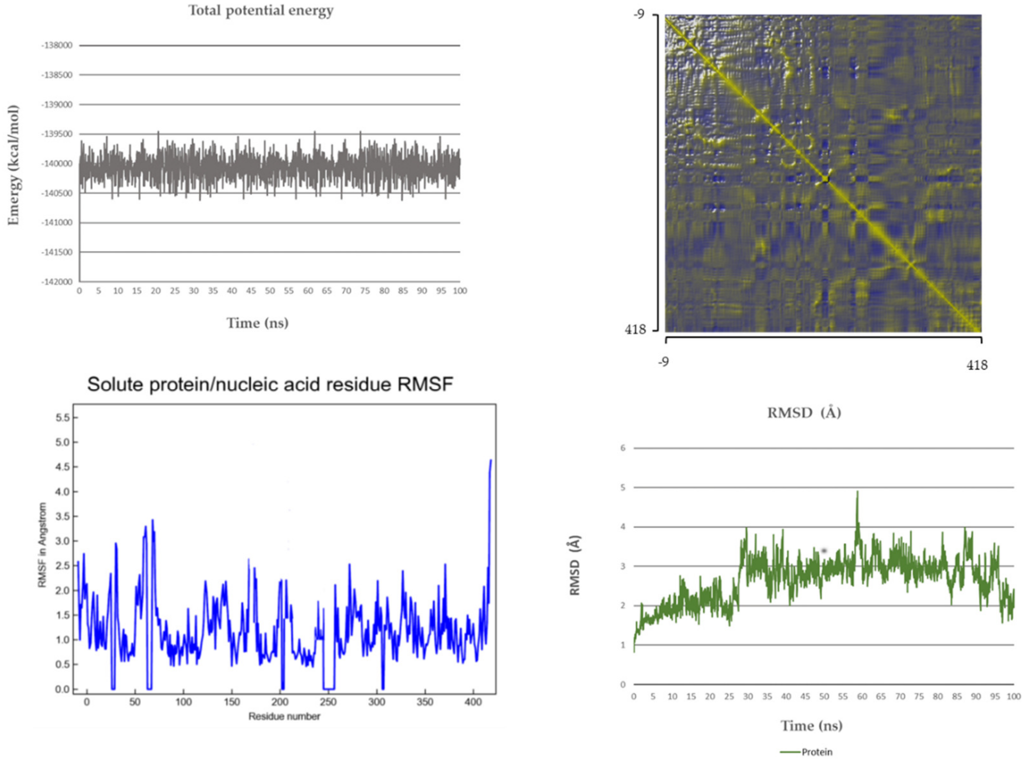

Figure S9. Total energy (up-left), DCCM (up-right), RMSF (down-left) and RMSD (down-right) of BoNT/A Light Chain in the free state.

Table S3. Structures and calculated free energies of binding (ΔG_B, in kcal/mol) of the selected compounds **1**–**10** for the LC serotypes B, C, D, E, F, and G

ID Compound	ΔG_B LC/B (PDB ID: 1F82)	ΔG_B LC/C (PDB ID: 2QN0)	ΔG_B LC/D (PDB ID: 2FPQ)	ΔG_B LC/E (PDB ID: 1T3A)	ΔG_B LC/F (PDB ID: 2A8A)	ΔG_B LC/G (PDB ID: 1ZB7)
ZINC000005008970	—	−7.3	—	−7.2	—	−8.2
ZINC000005008966	—	—	—	—	—	−8.6
ZINC000053720402	—	—	—	—	—	−7.3
ZINC000005729284	−7.5	−8.7	−7.3	−7.7	−8.2	−7.0
ZINC000053720401	—	—	—	−6.6	—	−8.5
ZINC000000000012	−6.7	—	—	—	—	−8.2
ZINC000005008967	—	−8.0	—	—	—	—
MolPort-003-757-821	−7.4	—	—	—	—	—
MolPort-002-518-389	—	—	—	—	—	−8.3
SN00068206	−6.9	—	—	—	—	—

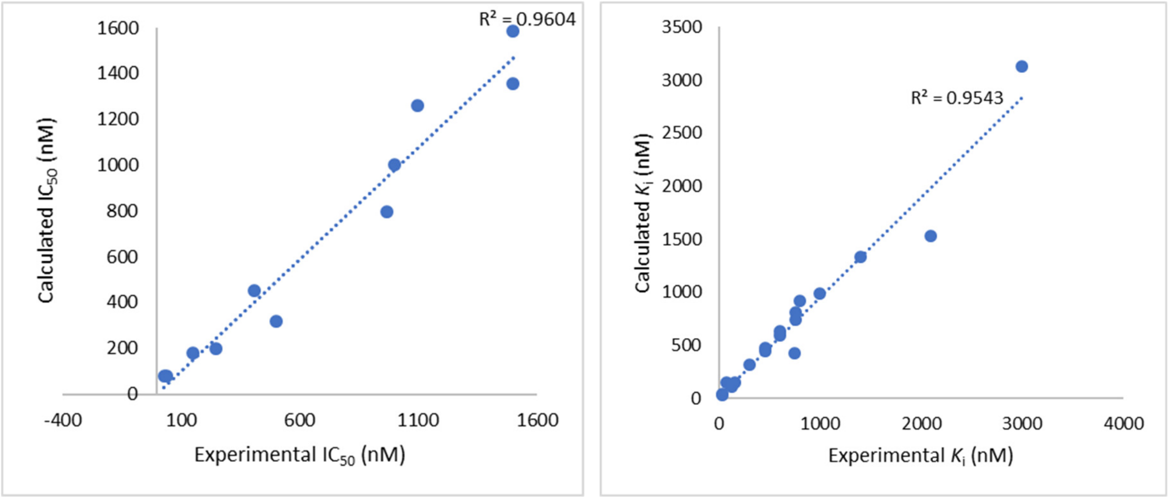

Figure S10. Linear regression plots and correlation coefficients of calculated *vs*. experimental IC_{50} (left) and K_i (right) values obtained for CONTROL dataset. Values from Table S1.

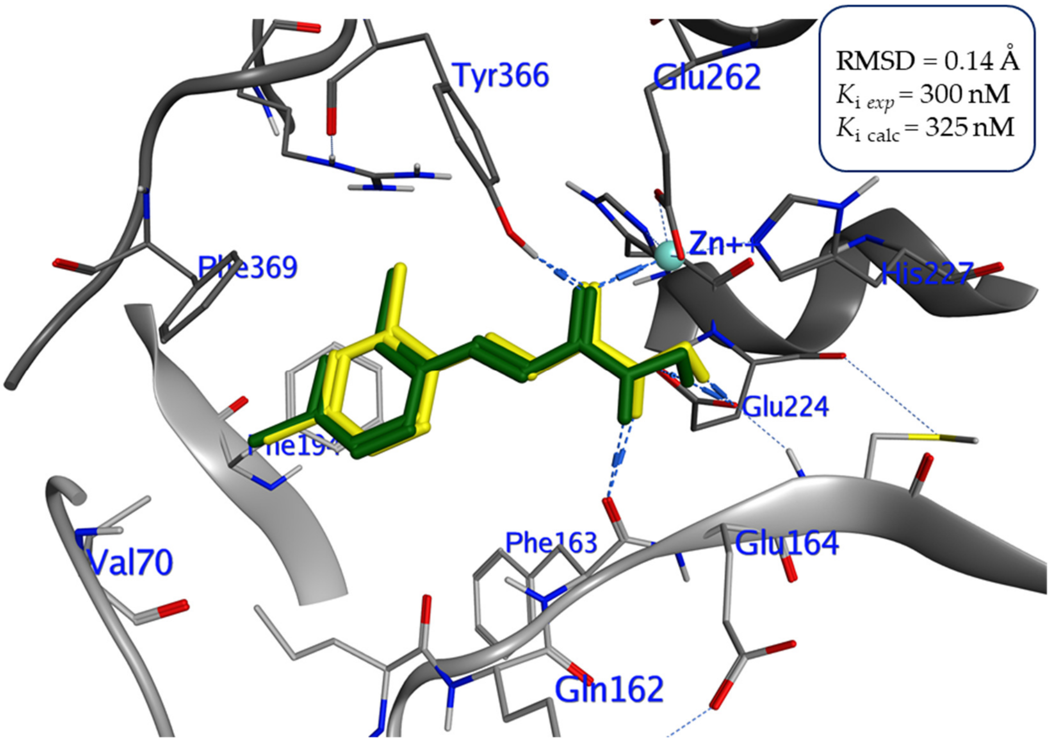

Figure S11. Crystallized laying (yellow) and docked pose (green) of (2*E*)-3-(2,4-dichlorophenyl)-*N*-hydroxyacrylamide inside the binding pocket with the respective values of root mean square (RMSD) and experimental and predicted K_i values.

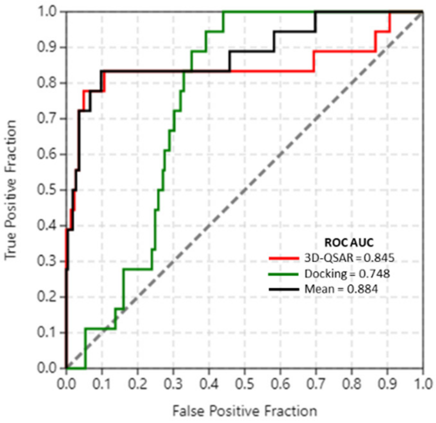

Figure S12. ROC curves for the scoring functions.

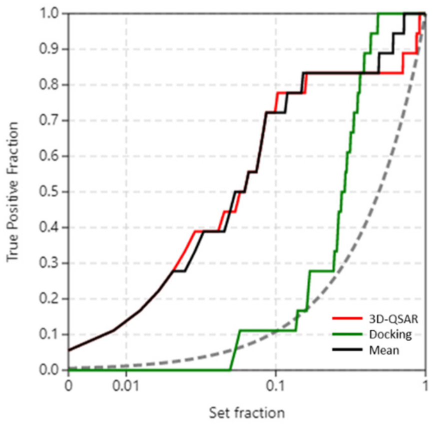

Figure S13. Enrichment curves for the scoring functions.

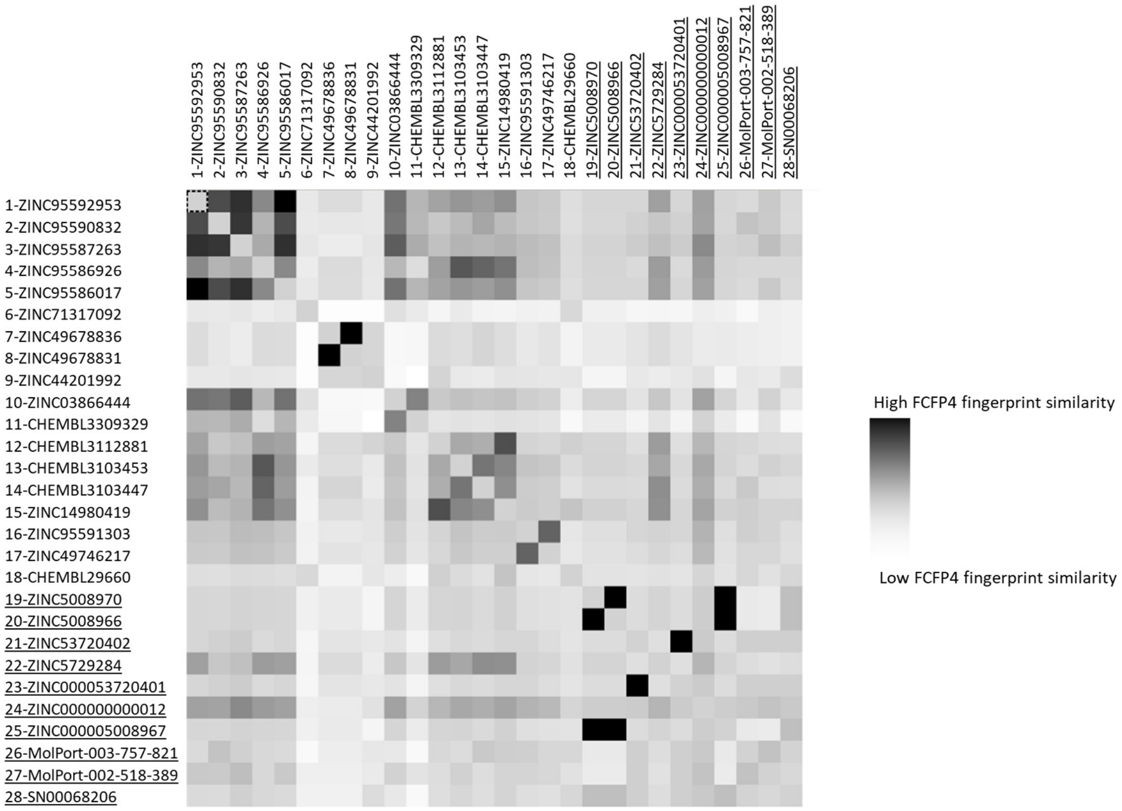

Figure S14. FCFP4 fingerprint similarity matrix.
